# Supplementary material for: Ferroelectricity driven magnetism at domain walls in LaAlO3/PbTiO3 superlattices
Source: Sci Rep. 2015 Aug 13;5:13052. doi: 10.1038/srep13052 (PMC4642506; doi:10.1038/srep13052)
Supplement: Supplementary Information [file srep13052-s1.doc]

Supplementary Material of

**Ferroelectricity driven magnetism at domain walls in LaAlO_3_/PbTiO_3_ superlattices**

P. X. Zhou^1,2^, S. Dong^3^,^a)^ H. M. Liu^1^, C. Y. Ma^1^, Z. B. Yan^1^, C. G. Zhong^2^ and J. -M. Liu1 [[1]](#footnote-2)b)

^1^*Laboratory of Solid State Microstructures and Collaborative Innovation Center of Advanced Microstructures, Nanjing University, Nanjing 210093, China*

^2^ *School of Science, Nantong University, Nantong 226007, China*

*^3^Department of Physics, Southeast University, Nanjing 211189, China*

**Comparison of GGA, GGA+*U*, LDA, LDA+*U*, and HSE**

All calculations in the article, including the SLs' structures relaxing, use the pure GGA or GGA+*U* method. In the supplementary, the different approaches are compared to check the reliability.

First, the values of relaxed PbTiO_3_ (PTO) and LaAlO_3_ (LAO) structures are listed in Table I to compare with the experimental values. For both PTO and LAO, the GGA and GGA+*U* methods always give larger lattices than those obtained using the LDA and LDA+ *U* methods. For the cubic LAO, the GGA method is the best choice according to the structural calculations, while the LDA and LDA+ *U* methods are not accurate. For the tetragonal PTO in the FE state, the GGA method gives the best in-plane lattice constant but a too long *c* axis, in agreement with previous studies^1^. The GGA+*U* method gives the best out-of-plane lattice constant, but the predicted in-plane one is a little larger.

Second, to confirm the reliability of our above results, we have re-done the relaxation for the most interesting *m*=4.5 SL using the GGA+*U* method. As listed in Table. III, the GGA+ *U* and pure GGA methods give the PTO structures in the two limits: small *vs* large *c*/*a* ratio. Then the real PTO should be within these two limits. The substrate is fixed to be the experimental LAO (3.79 Å at room-temperature). Although the relaxed structure is not identical to the above pure GGA one, the asymmetric FE domain and its affiliated magnetism remain alive, as shown in Fig.1 The opponent one, namely the symmetric FE domain, always exists regardless of the method or thickness. Therefore, our physical conclusion, i.e. the switchable FE domains and domain wall carried magnetism, remain robustly reliable, which should be insensitive to the details of technique.

**TABLE I.** Lattice constants relaxed using different approaches. Some typical experimental (EXP) values are also listed for comparison. Here the values of *U* and *J* are the same as those used in the main text. The band gaps (in unit of eV) are also shown for comparison.

| Material | Method | *a* | *c* | *a*/*c* | gap |
| --- | --- | --- | --- | --- | --- |
| PTO(FE) | GGA | 3.905 | 4.635 | 1.187 | 2.0 |
|  | GGA+*U* | 3.990 | 4.077 | 1.022 | 2.3 |
|  | LDA | 3.865 | 4.038 | 1.045 | 1.7 |
|  | LDA+*U* | 3.913 | 3.952 | 1.010 | 2.0 |
|  | HSE | 3.844 | 4.240 | 1.103 | 3.1 |
|  | EXP(16K)^2^ | 3.891 | 4.168 | 1.071 |  |
|  | EXP(300K)^3^ | 3.904 | 4.158 | 1.065 |  |
|  | EXP^4^ |  |  |  | 3.6 |
| PTO(PE) | GGA | 3.971 |  |  | 1.8 |
|  | GGA+*U* | 4.004 |  |  | 2.4 |
|  | LDA | 3.891 |  |  | 1.7 |
|  | LDA+*U* | 3.922 |  |  | 2.1 |
|  | HSE | 3.900 |  |  | 3.2 |
|  | EXP(800K)^3^ | 3.969 |  |  |  |
| LAO | GGA | 3.811 |  |  | 3.8 |
|  | GGA+*U* | 3.839 |  |  | 3.7 |
|  | LDA | 3.740 |  |  | 3.6 |
|  | LDA+*U* | 3.769 |  |  | 3.6 |
|  | HSE | 3.754 |  |  | 4.8 |
|  | EXP^5^ | 3.789 |  |  | 5.6 |


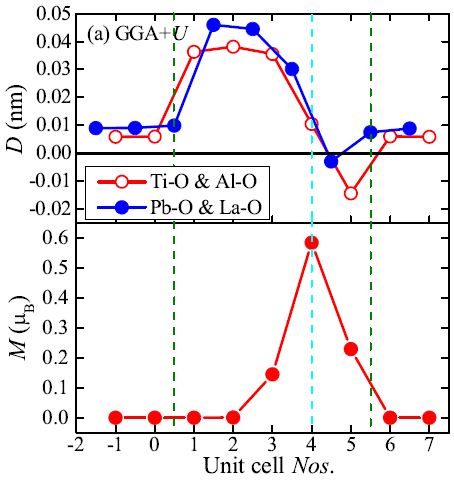


**Fig.S1.** Results of the *m*=4.5 SL with *n*-*n* type interfaces, calculated with relaxed structure using the full GGA+*U* method. (a) The local displacements between anions and cations. The vertical olive dash lines and cyan dot lines denote the interfaces and domain walls, respectively. (b) Local magnetic moments.

***References:***

1. Wang, Y., Niranjan, M. K., Jaswal, S. S. & Tsymbal, E. Y. First-principles studies of a two-dimensional electron gas at the interface in ferroelectric oxide heterostructures. *Phys. Rev. B* **80**, 165130 (2009).
2. Schönau, K. A., Knapp, M., Ehrenberg, H., Meštrić, H., Eichel, R.-A. & Fuess, H. “Low-temperature high-resolution measurement of PbTiO_3_ in combination with ERP,”

http://hasyweb.desy.de/science/annual reports/2004 report/part1/contrib/41/11430.

1. Kuroiwa, Y., Aoyagi, S., Sawada, A., Harada, J., Nishibori, E., Takata, M. & Sakata, M. Evidence for Pb-O covalency in tetragonal PbTiO_3_ *Phys. Rev. Lett.* **87**, 217601 (2001).
2. Moret, M. P., Devillers, M. A. C., Wörhoff, K. & Larsen, P. K. Optical properties of PbTiO_3_, PbZr*_x_*Ti_1−_*_x_*O_3_, and PbZrO_3_ films deposited by metalorganic chemical vapor on SrTiO_3_*. J. Appl. Phys.* **92**, 468 (2002).
3. Ohtomo A. and Hwang, H. Y. A high-mobility electron gas at the LaAlO_3_/SrTiO_3_ heterointerface. *Nature* (*London*) **427**, 423-426 (2004).

1. ^ba)^ Corresponding author, E-mail: [sdong@seu.edu.cn](mailto:sdong@seu.edu.cn)

   ^b)^ Corresponding author, E-mail: [liujm@nju.edu.cn](mailto:liujm@nju.edu.cn) [↑](#footnote-ref-2)
